# Real-world data on clinical outcomes and validation of prognostic models for angioimmunoblastic T-cell lymphoma: a multicentric retrospective study in Southern China

**DOI:** 10.3389/fonc.2025.1580370

**Published:** 2025-06-10

**Authors:** Qiu-Yuan Xiang, Jing-Song Wu, Ling Huang, Zong-Kai Zou, Ting-Bo Liu, Zhi-Gang Peng, Li-E. Lin, Xiao-Lei Wei, Hong-Yu Zhang, Yun Lin, Guo-Wei Li, Yi-Rong Jiang, Hua Wang, Ke-Qian Shi, Rui-Ji Zheng, Zhi-Jun Han, Xiao Qiu, Wen-Yu Li, Ji-Hao Zhou

**Affiliations:** ^1^ Department of Hematology, The Second Clinical Medical College of Jinan University, Shenzhen, China; ^2^ Department of Lymphoma, Guangdong Provincial People’s Hospital, Guangdong Academy of Medicine Sciences, Southern Medial University, Guangzhou, China; ^3^ Department of Pathology, Zhangzhou Affiliated Hospital of Fujian Medical University, Zhangzhou, Fujian, China; ^4^ Department of Hematology, Fujian Institute of Hematology, Fujian Provincial Key Laboratory of Hematology, Fujian Medical University Union Hospital, Fuzhou, China; ^5^ Department of Hematology, First Affiliated Hospital of Guangxi Medical University, Nanning, China; ^6^ Department of Hematology, Hainan General Hospital, Hainan Affiliated Hospital of Hainan Medical University, Haikou, China; ^7^ Department of Hematology, Nanfang Hospital and Southern Medical University, Guangzhou, China; ^8^ Department of Hematology, Peking University Shenzhen Hospital, Shenzhen, China; ^9^ Department of Hematology, Fujian Provincial Hospital, Fuzhou, China; ^10^ Department of Hematology, Huizhou Central People’s Hospital, Huizhou, China; ^11^ Department of Hematology, Affiliated Dongguan People’s Hospital, Southern Medical University (Dongguan People’s Hospital), Dongguan, China; ^12^ Department of Hematology, State Key Laboratory of Oncology in South China, Guangdong Provincial Clinical Research Center for Cancer, Sun Yat-Sen University Cancer Center, Guangzhou, China; ^13^ Department of Hematology, The First People’s Hospital of Yunnan Province, Kunming, China; ^14^ Department of Hematology, Zhangzhou Affiliated Hospital of Fujian Medical University, Zhangzhou, Fujian, China; ^15^ Department of Hematology, Shenzhen People’s Hospital (The Second Clinical Medical College of Jinan University; The First Affiliated Hospital of Southern University of Science and Technology), Shenzhen, China; ^16^ Department of Hematology, Guangdong Provincial People’s Hospital, Guangdong Academy of Medical Sciences, Southern Medical University, Guangzhou, China

**Keywords:** angioimmunoblastic T-cell lymphoma (AITL), prognostic model, clinical characteristics, survival rate, real-world study, South China AITL score

## Abstract

**Background:**

This study aimed to elucidate the treatment outcomes and prognosis of angioimmunoblastic T-cell lymphoma (AITL) patients in a real-world setting.

**Objectives:**

The clinical efficacy of new drug applications was evaluated, alongside the predictive accuracy of prognostic models, to inform future research.

**Methods:**

In this study, 82.9% of patients received a CHOP-like regimen, while 36.4% also received chidamide. We assessed the prognostic models’ predictive power using the Cox proportional hazards model and concordance index (C-index).

**Results:**

The median age of the patients in this study was 62.0 years, with 2-year progression-free survival (PFS) and overall survival (OS) rates of 36.1% and 60.3%, respectively. Complete response (CR) rates in first-line treatments were 21.6% for the chidamide-containing group and 28.1% for the chidamide-free group. The AITL scores, PIAI scores, and Chinese AITL scores demonstrated superior C-index values, with the Chinese AITL score providing the most distinct risk stratification. Advanced age (over 70 years), bone marrow involvement, and CD7 negativity were identified as significant prognostic factors associated with poorer PFS in both univariate and multivariate analyses. A novel prognostic model, the South China AITL Score, was constructed by combining these three factors, stratifying patients into low-risk and high-risk groups, with 5-year PFS rates of 81.5% and 34.6%, respectively. This model was successfully validated in an independent cohort.

**Conclusions:**

The prognosis of AITL in real-world settings is poor, and the addition of chidamide did not show improvement in remission rates or survival. Our novel prognostic model, along with the Chinese AITL score, may enhance the identification of Chinese patients at varying risks for chemotherapy. Furthermore, the pathological marker CD7 is anticipated to emerge as a significant biomarker for the prognostic evaluation of AITL.

## 1 Introduction

Angioimmunoblastic T-cell lymphoma (AITL) is a distinctive subtype of peripheral T-cell lymphoma (PTCL) that originates from follicular helper T (TFH) cells.

The epidemiological distribution of AITL exhibits notable geographical heterogeneity. Globally, AITL constitutes 28.7% of PTCL cases in Europe, 17.9% in Asia, and 16.0% in North America (25). In China, AITL represents 13.8% of all PTCL diagnoses (1). TFH cells are a CD4+ helper T subset that play a vital role in promoting the generation of plasma cells and memory B cells. Dysfunction of TFH cells may lead to disruptions within germinal centers, ultimately leading to the development of AITL (2).

The pathogenesis of AITL is thought to involve complex processes involving multiple hits. The first hit occurs at an early stage of hematopoietic development, when mutations occur in the TET2 and DNMT3A genes. The second hit occurs at the mature T-cell stage, when cells with mutated genes suffer further mutations in the RHOA and/or IDH2 gene. These later mutations are instrumental in modulating the differentiation and proliferation of clonal TFH cells, thereby facilitating the occurrence and progression of AITL (3–9).

AITL is observed mostly in the elderly population. The clinical presentation is typically manifested as a systemic illness, including widespread lymphadenopathy, B symptoms and splenomegaly, as well as bone marrow involvement. Additionally, patients may exhibit plasma effusions, pruritus, or rashes (10). AITL is often characterized by an aggressive disease course. Most patients are diagnosed with advanced disease. The standard first-line therapy is the CHOP regimen, which comprises cyclophosphamide, doxorubicin, vincristine, and prednisone. Nevertheless, the 5-year overall survival (OS) rate for AITL patients remains below 50% (11, 12).

To enhance the therapeutic efficacy for patients with newly diagnosed AITL, researchers have attempted to combine novel agents with CHOP in strategies termed “CHOP plus X.” These novel agents include epigenetic modulators, immune checkpoint inhibitors, and signal transduction inhibitors. The combination of romidepsin or brentuximab vedotin (BV) with CHOP failed to improve the long-term prognosis of patients with AITL (13, 14), whereas a phase II clinical trial demonstrated that the combination of CHOP with chidamide had a better overall response rate (ORR) of 50% and a complete remission (CR) rate of 40% in patients with AITL, with a more sustained duration of response (DOR) (15). Another study focusing on PTCL also showed that patients treated with chidamide plus chemotherapy had a longer progression-free survival (PFS) than those receiving conventional chemotherapy. Unfortunately, the study did not find a statistically significant divergence in OS between the two treatment groups (16). A recent large-scale, multicenter, retrospective study showed that patients who underwent autologous hematopoietic stem cell transplantation (ASCT) achieved 5-year OS and PFS rates of 50.8% and 39.4%, respectively. For AITL patients specifically, the 5-year PFS rate was 47.3%, substantiating the significant survival benefits conferred by ASCT (17). However, few Chinese patients have undergone ASCT in a real-world setting.

Despite the availability of numerous prognostic models, most of the studies are based on retrospective data from PTCL or non-Hodgkin lymphoma (NHL) patients rather than from AITL patients. Furthermore, the patients enrolled in clinical trials may not accurately represent the real-world population. Besides, it is unclear which prognostic model is most appropriate for Chinese patients. This study was conducted using data from the South China Cooperative Group with the aims of elucidating the treatment outcomes and prognosis of AITL patients in a real-world setting, assessing the clinical value of new drug applications, evaluating the predictive power of various prognostic models, and offering valuable insights for future research endeavors.

## 2 Methods

### 2.1 Study design and patients

A total of 140 patients with newly diagnosed AITL were enrolled retrospectively from 19 lymphoma centers between May 2014 and May 2022 by the South China T-cell Lymphoma Collaborative Group. All patients fulfilled the 2016 World Health Organization (WHO) diagnostic criteria for AITL (18) and underwent imaging and bone marrow examinations to determine the clinical stage. All patients enrolled met the following inclusion criteria: complete clinical follow-up data and a confirmed initial diagnosis of AITL by pathology examinations. The exclusion criteria were as follows: 1) AITL patients who were in relapse at the time of enrollment; 2) AITL patients without available clinical follow-up data; 3) AITL patients concurrent with other malignancies; and 4) patients who did not receive treatment. We collected the clinical data, laboratory test results, and imaging examination results of all enrolled patients. This study was approved by the Ethics Committee of Guangdong Provincial People’s Hospital, with the ethical review number:KY-Q-2021-225-02, and all research subjects signed informed consents.

### 2.2 Criteria for response evaluation

For patients who underwent interim or end-of-treatment response evaluation by positron emission tomography/computed tomography (PET/CT), the Deauville 5-point scale and Cheson criteria were applied to evaluate treatment response (19, 20).

### 2.3 Prognostic models

In this study, we compared seven prognostic models that were developed for risk stratification in lymphoma patients: the International Prognostic Index (IPI), the Peripheral T-cell Lymphoma Prognostic Index (PIT), the National Comprehensive Cancer Network International Prognostic Index (NCCN-IPI), the Angioimmunoblastic T-cell Lymphoma Prognostic Index (ATPI), the Alternative Prognostic Indicators for AITL (PIAI), the Prognostic Index for AITL score, and the Chinese Angioimmunoblastic T-cell Lymphoma Index (Chinese AITL score) (10, 11, 21–25). All patients were stratified for prognostic risk based on a set of clinical variables, such as age, Eastern Cooperative Oncology Group performance status (ECOG PS), and Ann Arbor stage, as well as laboratory variables, including hemoglobin, lactate dehydrogenase (LDH), and albumin.

### 2.4 Statistical analysis

OS was defined as the time from the date of diagnosis to either the date of death from any cause or the date of last follow-up. PFS was defined as the time from diagnosis to disease relapse or progression or to the last follow-up. The Kaplan–Meier method was used for survival analysis, while the log-rank test was used to evaluate the significant differences between groups. Multivariate analyses were performed using the Cox proportional hazard regression model. The concordance index (C-index) was utilized to assess and compare the predictive power of the prognostic models; a C-index of 0.5 indicates no predictive ability, whereas a value of 1 reflects perfect concordance between the model’s predictions and observed outcomes. A higher C-index signifies superior discriminatory power (26, 27). All the statistical tests were two-tailed, and P < 0.05 was statistically significant. Data processing and analysis were performed using SPSS version 25.0 and EZR software.

## 3 Results

### 3.1 Clinical characteristics

The demographic characteristics of the 140 enrolled patients are detailed in **Table 1**, with 85 males (60.7%) and 55 females (39.3%). The median age was 62 years (17-83 years), and 55.0% were aged 60 or older, with 15.0% over 70. At diagnosis, 95.0% had stage III or IV disease. Bone marrow involvement was present in 19.3%, and extranodal involvement at multiple sites in 20.7%. The most commonly affected extranodal sites were the bone marrow (19.3%), nasal cavity (15.0%), and lung (10.0%).

**Table 1:** AITL clinicodemographic characteristics(n=140).

Variable	No. (%)
Age years	
Median (range)	62.0 (17-83)
>60	77/140 (55)
>70	21/140 (15)
Sex	
male	85/140 (60.7)
female	55/140 (39.3)
Ann Arbor stage	
I	1/140 (0.7)
II	6/140 (4.3)
III	53/140 (37.9)
IV	80/140 (57.1)
LDH>240 U/L	85/138 (61.6)
Aalbumin level<35g/L	78/137 (56.9)
IgA level<400mg/dL	35/45 (77.8)
CRP≥10mg/L	51/80 (63.7)
β2-MG≥2.4mh/L	81/100 (81)
Anemia (Hb<100g/L)	101/139 (72.7)
Ki-67>60%	42/125 (33.6)
Positive HBsAg	14/124 (11.3)
Positive HCV	2/120 (1.7)
Positive EBER	64/101 (63.7)
Positive EBV-DNA	27/105 (25.7)
Median	400
Average	747.7
NO.of extranodal sites≥2	29/140 (20.7)
Extranodal sites	
bone marrow	27/140 (19.3)
Nose	21/140 (15.0)
Lungs	14/140 (10.0)
Parotid gland	11/140 (7.9)
Bone	8/140 (5.7)
Liver	6/140 (4.3)
Skin	5/140 (3.6)

LDH, lactate dehydrogenase; CRP, C-reactive protein; β2-MG, β2-macroglobulin; HBsAg, Hepatitis B surface antigen; HCV, Hepatitis C virus.

### 3.2 Pathological characteristics

AITL is distinguished by a distinctive follicular architecture marked by clusters of TFH-associated markers, including CD4, CD7, PD-1and CD10 (28). In this study, immunohistochemistry revealed the following positivity rates: CD10 (63.3%), CD30 (78.2%), CD7 (80.28%), and Ki-67 (33.6% for values exceeding 60%). (**Supplementary Table S1**).

### 3.3 Treatment and responses

**Figure 1:**
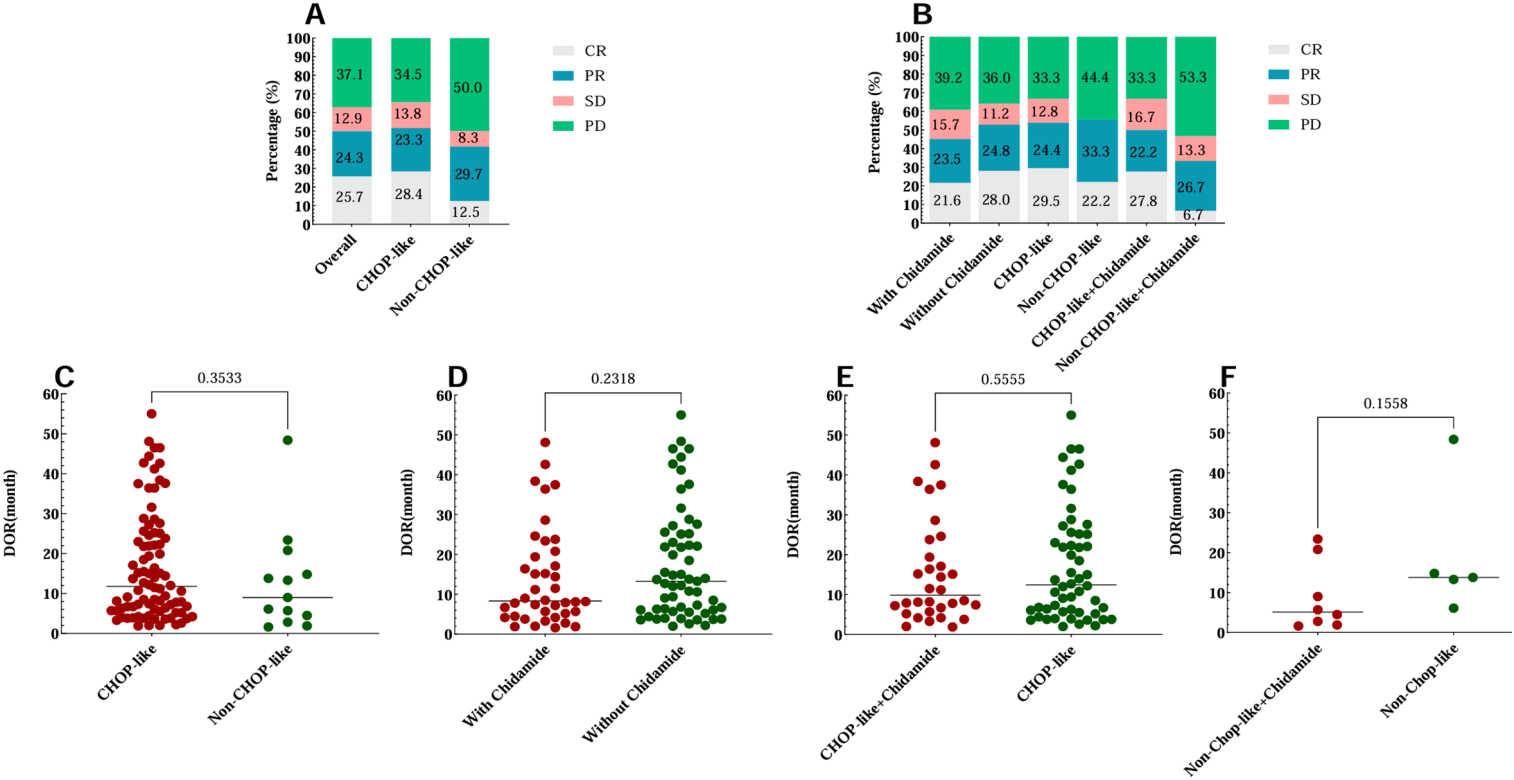
First-line treatment efficacy and DOR of 140 AITL patients. **(A)** Treatment efficacy for patients treated with CHOP-like regimens and non-CHOP-like regimens. **(B)** Treatment efficacy for patients treated with different regimens with or without chidamide combined with CHOP-like regimens or non-CHOP-like regimens. **(C-F)** The DOR of AITL patients who received different treatment regimens. DOR, duration of remission.

Among the 140 patients, 116 were initially treated with CHOP-like regimens and 24 with non-CHOP-like regimens, which comprised 13 cases of chidamide monotherapy, one of gemcitabine monotherapy, and seven combination therapies including gemcitabine (GDP or P-Gemox). Comparative baseline analysis revealed a significant difference in the proportion of patients aged 70 or older between the CHOP-like and non-CHOP-like treatment groups (P < 0.05). No significant differences were found in demographics and laboratory parameters such as sex, age, white blood cell count, hemoglobin, platelet count, LDH, C-reactive protein (CRP), Ki-67 index over 60%, β2-MG, albumin, ECOG PS, bone marrow involvement, Ann Arbor stage, or extranodal disease (**Supplementary Table S2**). In first-line treatment, chidamide was used in 51 patients (36.4%) and BV in 6 patients (4.3%) of the total cohort. Among 140 patients evaluated for treatment efficacy, the overall response distribution was as follows: CR in 36 cases (25.7%; 95% CI 18.7-33.8), partial response (PR) in 34 cases (24.3%; 95% CI 17.4-32.3), stable disease (SD) in 18 cases (12.9%; 95% CI 7.8-19.6), and progressive disease (PD) in 52 cases (37.1%; 95% CI 29.1-45.7). The ORR reached 50.0% (95% CI 41.4-58.6). In first-line treatment analysis, CHOP-like regimens demonstrated a CR rate of 28.5% (95% CI 20.5-37.6) and PR rate of 23.3% (95% CI 15.9-32.0), yielding an ORR of 51.7% (95% CI 42.3-61.1). In contrast, non-CHOP-like regimens showed lower efficacy with CR and PR rates of 12.5% (95% CI 2.6-32.3) and 29.2% (95% CI 12.6-51.1) respectively, resulting in an ORR of 41.7% (95% CI 22.1-63.4). The CR and ORR were slightly higher with CHOP-like regimens, but this difference was not statistically significant (P=0.261) (**Figure 1A**). The median DOR was 495.4 ± 406.8 days for the CHOP-like group and 383.2 ± 384.8 days for the non-CHOP-like group, with no significant difference (P=0.353) (**Figure 1C**).

To evaluate the efficacy of chidamide in the first-line treatment of patients with AITL, participants were categorized into two groups: one receiving chidamide and a control group that did not. The baseline characteristics of these groups were found to be statistically similar, as detailed in **Supplementary Table S3**. The treatment outcomes are as follows: the chidamide-containing group exhibited a CR rate of 21.6% (95% CI 11.3-35.3), while the chidamide-free group showed a CR rate of 28.1% (95% CI 19.1-38.6). PR rates were 23.5% (95% CI 12.8-37.5) and 24.7% (95% CI 16.2-35.0), respectively, and ORR were 45.1% (95% CI 31.1-59.7) and52.8% (95% CI 41.9-63.5) for the chidamide-containing and chidamide-free groups, respectively. The addition of chidamide did not significantly improve remission rates, as depicted in **Figure 1B**. Among responders, the DOR for those treated with chidamide was approximately 14.04 months ± 12.43 months, compared to 18.03 months ± 14.07 months for those not treated with chidamide (P = 0.230). When chidamide was administered in conjunction with CHOP-like regimens, the DOR was 15.37 months ± 13.0 months in the chidamide group, as opposed to 17.19 months ± 13.97 months without chidamide (P = 0.553). For non-CHOP-like regimens, the DOR in the chidamide group was 8.71 months ± 8.62 months, compared to 19.27 months ± 16.66 months without chidamide (P = 0.156). These findings are further illustrated in **Figures 1D-F**.

Maintenance therapy was administered to 19 patients (13.6%) as part of the first-line treatment strategy, with 11 (57.9%) receiving chidamide, 3 (15.8%) on interferon alpha, 1 on BV, and 4 on thalidomide or lenalidomide. Patients receiving maintenance therapy did not exhibit a prolonged DOR. Eight patients (5.7%) underwent ASCT as part of their first-line treatment, with 6 at complete response 1 (CR1) and 2 at partial response 1 (PR1). The DOR for those who received ASCT was 15.93 months ± 6.37 months, compared to 15.65 months ± 13.48 months for others (P = 0.927). The lack of observed DOR extension with ASCT consolidation is likely attributed to the small sample size.

### 3.4 Survival outcome

**Figure 2:**
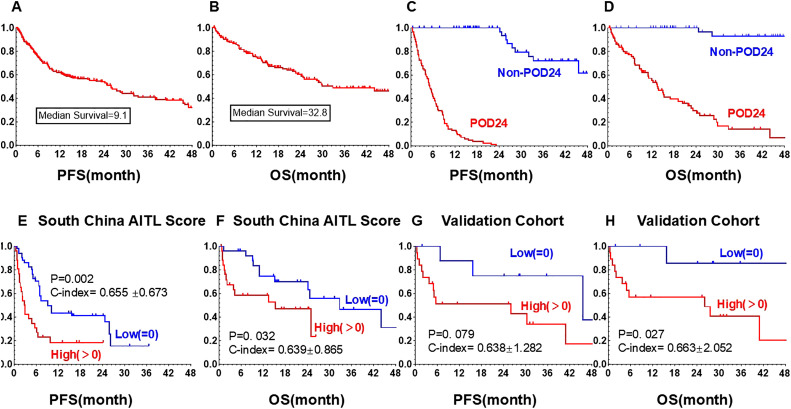
Kaplan-Meier curves of PFS and OS. **(A)** PFS of all 140 AITL patients. **(B)** OS of all 140 AITL patients. **(C)** PFS of the POD24 group and non-POD24 group. **(D)** OS of patients in the POD24 group and non-POD24 group. **(E)** PFS of South China AITL Score patients in the low-risk group and high-risk group. **(F)** OS of South China AITL Score patients in the low-risk group and high-risk group. **(G)** PFS of validation group patients in the low-risk group and high-risk group. **(H)** OS of validation group patients in the low-risk group and high-risk group. PFS, progression-free survival; OS, overall survival; POD24, PD within 24 months.

The median follow-up was 27.8 months (95% CI, 18.7 to 36.7). For the entire cohort, the 2-year PFS rate was 36.1%, and the median PFS was 9.1 months (95% CI, 6.2 to 12.0 months). The 2-year OS rate was 60.3%, and the median OS was 32.8 months (95% CI, 16.4 to 49.2 months) (**Figures 2A, B**). 85 patients (60.7%) experienced PD within 24 months (POD24). The median PFS for the POD24 group was significantly shorter, at 5.1 months (95% CI, 4.2 to 6.0 months), than that for the non-POD24 group, who had a median PFS of 49.2 months (95% CI, 42.8 to 55.6 months) (P < 0.001) (**Figure 2C**). The median OS for the POD24 group was 13.8 months (95% CI, 10.8 to 16.8 months), while the median OS for the non-POD24 group was not reached (P < 0.001) (**Figure 2D**). The POD24 group exhibited a significantly poorer prognosis than the non-POD24 group.

Both univariate and multivariate analysis confirmed that advanced age (over 70 years), bone marrow involvement, and CD7 negativity were significant prognostic factors for worse PFS (**Table 2**). Combining the three factors identified through multivariate analysis, we selected 53 complete cases that included these three indicators and constructed a novel prognostic model termed as “South China AITL Score.” Patients were categorized into two risk groups: the low-risk group with 0 adverse factors and the high-risk group with 1-3 adverse factors. In the South China AITL Score population, median PFS and OS were not attained in either low- or high-risk groups. The 5-year OS rates were 92.1% for the low-risk group and 58.5% for the high-risk group (p=0.032), and the corresponding PFS rates were 81.5% and 34.6%, respectively (p=0.002). The C-index for the South China AITL Score in predicting PFS was 0.655 ± 0.673 (**Figure 2E**), and for OS it was 0.639 ± 0.865 (**Figure 2F**).

**Table 2:** Univariate analysis for OS and PFS and multivariate analysis for OS.

Variable	PFS						OS					
	Univariate analysis			Multivariate analysis			Univariate analysis			Multivariate analysis		
	HR	95%CI	P	HR	95%CI	P	HR	95%CI	P	HR	95%CI	P
Sex, male	0.947	0.626-1.434	0.797				0.865	0.508-1.474	0.595			
Age>70years	0.440	0.258-0.752	**0.003**	0.360	0.152-0.851	**0.020**	0.429	0.226-0.815	**0.010**	0.407	0.142-1.171	0.095
Ann Arbor stage≥III	0.822	0.333-2.026	0.669				0.862	0269-2.766	0.803			
CRP≥10mg/l	0.596	0.336-1.055	0.075				0.616	0.296-1.281	0.194			
Extranodal site>1	0.746	0.464-1.256	0.288				0.854	0.429-1.702	0.654			
Bone marrow involvement	0.527	0.323-0.862	**0.011**	0.337	0.155-0.730	**0.006**	0.582	0.311-1.088	0.090	0.606	0.228-1.610	0.315
LDH>240 u/L	0.550	0.353-0.855	**0.008**	0.548	0.257-1.168	0.119	0.492	0.276-0.879	**0.017**	0.475	0.170-1.326	0.155
Albumin<35g/L	0.508	0.331-0.782	**0.002**	1.040	0.489-2.209	0.919	0.369	0.203-0.670	**0.001**	0.665	0.229-1.924	0.451
IgA>400mg/dL	1.064	0.402-2.816	0.900				0.939	0.263-3.352	0.922			
Ki-67index>60%	1.000	0.649-1.543	0.999				0.823	0.470-1.441	0.496			
CD30-positive	0.510	0.271-0.961	**0.037**	0.796	0.212-2.997	0.736	0.581	0.269-1.254	0.166	0.946	0.192-4.673	0.946
CD7-negative	0.346	0.177-0.677	**0.002**	0.344	0.147-0.801	**0.013**	0.309	0.126-0.759	**0.010**	0.434	0.151-1.249	0122

LDH, lactate dehydrogenase; CRP, C-reactive protein; OS, overall survival; PFS, progression free survival; 95% CI, 95% confidence interval.

Bold values denote statistical significance (P < 0.05).

The South China AITL Score model, validated in a cohort of 28 AITL patients from a single institution in China, demonstrated high predictive efficacy for PFS and OS, with C-index values of 0.638 ± 1.282 for PFS and0.663 ± 2.052 for OS, respectively (**Figures 2G, H**). The median PFS and OS were not reached in the low-risk group and high-risk group. The 5-year PFS rate and OS rate in the low-risk groups and high-risk groups was 37.5% vs. 17.1% (p = 0.079) and 42.9% vs.20.4% (p < 0.027).

### 3.5 Validation of different prognostic models

**Figure 3:**
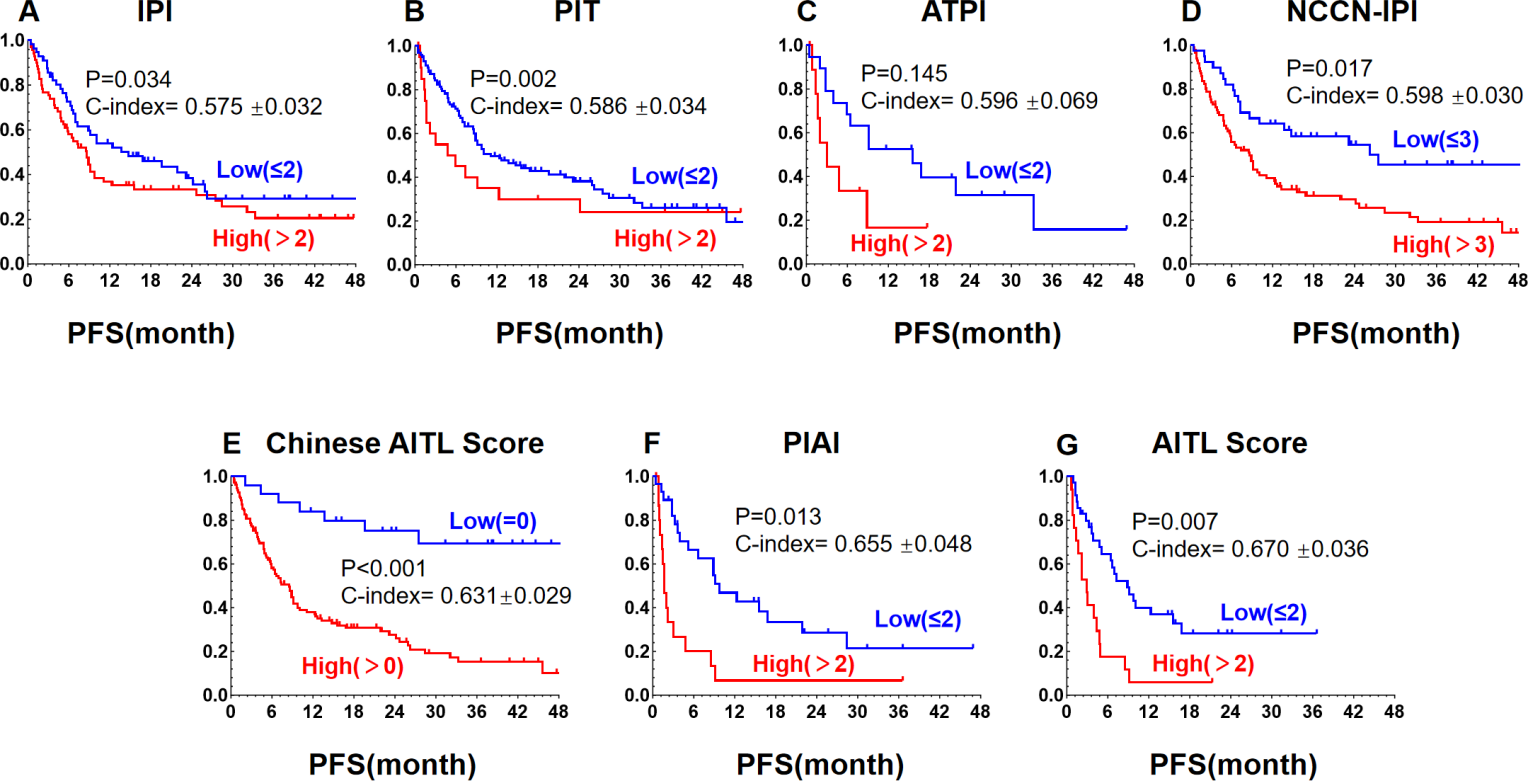
Kaplan-Meier curves for PFS in AITL patients stratified by different prognostic models: **(A)** International Prognostic Index (IPI), **(B)** Peripheral T-cell Lymphoma Prognostic Index (PIT), **(C)** Angioimmunoblastic T-cell Lymphoma Prognostic Index (ATPI), **(D)** National Comprehensive Cancer Network International Prognostic Index (NCCN-IPI), **(E)** Chinese Angioimmunoblastic T-cell Lymphoma Index (Chinese AITL score), **(F)** Alternative Prognostic Indicators for AITL (PIAI), **(G)** Prognostic Index for AITL score (AITL Score).

**Figure 4:**
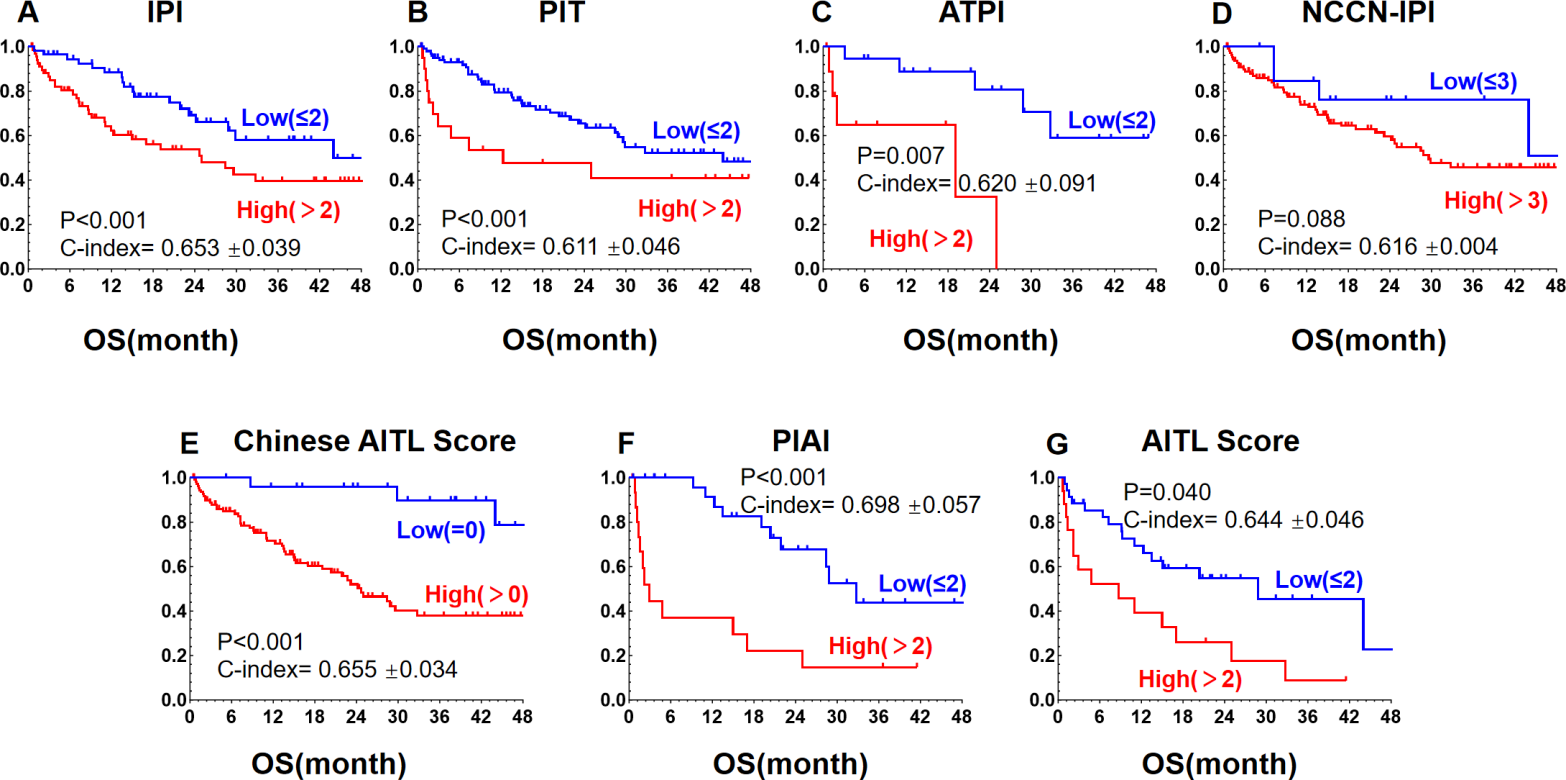
Kaplan-Meier curves for OS in AITL patients stratified by different prognostic models: **(A)** International Prognostic Index (IPI), **(B)** Peripheral T-cell Lymphoma Prognostic Index (PIT), **(C)** Angioimmunoblastic T-cell Lymphoma Prognostic Index (ATPI), **(D)** National Comprehensive Cancer Network International Prognostic Index (NCCN-IPI), **(E)** Chinese Angioimmunoblastic T-cell Lymphoma Index (Chinese AITL score), **(F)** Alternative Prognostic Indicators for AITL (PIAI), **(G)** Prognostic Index for AITL score (AITL Score).The revised manuscript (with updated figures and captions) has been re-uploaded. We appreciate your diligence and regret the inconvenience.

We further evaluated seven prognostic models developed for the risk stratification of lymphoma patients for comparative analysis. we scored our patients using the original criteria and divided them into high- and low-risk groups. **Figures 3** and **4** demonstrate that all models had good predictive power for both PFS and OS. The predictive efficacy was quantified using the C-index. For PFS, the top models were the AITL score (0.670 ± 0.036), PIAI (0.655 ± 0.048), and Chinese AITL score (0.631 ± 0.029). For OS, they were PIAI (0.698 ± 0.057), Chinese AITL score (0.655 ± 0.034), and AITL score (0.644 ± 0.046). **Table 3** presents the 5-year OS and PFS estimates from the original models and our calculated 1- to 4-year ranges. The Chinese AITL score showed exceptional predictive efficacy for both OS and PFS, with significant survival differences between the risk groups it defined (4-year PFS: 37.9% vs. 78.6%, 4-year OS: 10.1% vs. 69.3%, p < 0.001). Therefore, we recommend the Chinese AITL score as a suitable prognostic model for Chinese AITL patients.

**Table 3:** Risk stratification in lymphoma patients using seven prognostic models.

Model (N of patients)		Original study			Current study N=140		
	Model variables	Risk groups(point)	Years -OS%	Years -PFS (%)	Risk groups (point)	Years -OS%	Years -PFS (%)
IPI N=2031	Age>60 years	Low risk (0-1)	5Y-73.0	5Y-70.0			
	ECOG PS>1	Low-intermediate (2-3)	5Y-51.0	5Y-50.0	Low risk (≤2)	4Y-49.8	4Y-29.0
	Stage III/IV	High-intermediate (4-5)	5Y-43.0	5Y-49.0			
	EN sites>1	High risk (≥6)	5Y-26.0	5Y-40.0	High risk (>2)	4Y-39.7	4Y-20.5
	LDH>ULN						
PIAI N=243	Age>60						
	ECOG PS>1	Low risk (0-1)	5Y-44.0	5Y-28.0	Low risk (≤2)	3Y-43.9	3Y-21.5
	EN Sites≥1						
	Presence of B symptoms	High risk (2-4)	5Y-24.0	5Y-15.0	High risk (>2)	3Y-14.8	3Y-6.7
	PLT<15×10*9						
ATPI N=207	Age>60	Low risk (0-1)	3Y-85.0	5Y-73.0			
	EN Sites>1	Low-intermediate (2)	3Y-62.0	5Y-37.0	Low risk (≤2)	2Y-80.7	1Y-52.6
	PLT<15×10*9/L	High-intermediate (3)	5Y-51.0	5Y-36.0			
	WBC>1×10*12/L	High risk (4-6)	5Y-12.0	5Y-13.0	High risk (>2)	2Y-32.4	1Y-16.7
	Hb<100 g/L						
	IgA>400mg/dl						
AITL score N=282	Age≥60 years	Low risk (0-1)	5Y-63.0	5Y-41.0	Low risk (≤2)	3Y-45.6	1Y-36.9
	ECOG PS>2	Intermediate- risk (2)	5Y-54.0	5Y-37.0			
	CRP>ULN	High risk (3-4)	5Y-21.0	5Y-13.0	High risk (>2)	3Y-8.6	1Y-5.9
	Presence of B symptoms						
Chinese AITL score N=164	Age>70 years	Low risk (0)	5Y-69.0	NA			
	LDH>ULN	Intermediate risk (1)	5Y-41.5	NA	Low risk (≤0)	4Y-78.6	4Y-69.3
	Albumin<35g/L	High risk (2-3)	5Y-23.7	NA	High risk (>0)	4Y-37.9	4Y-10.1
NCCN-IPI N=1650	Age, years(>40, <60; ≥60, ≤75; >75)	Low risk (0-1)	5Y-96.0	5Y-91.0	Low risk (≤3)	4Y-58.9	4Y-45.4
	ECOG PS≥2	Low-intermediate (2-3)	5Y-82.0	5Y-74.0			
	LDH(>1, ≤3; >3)	High-intermediate (4-5)	5Y-64.0	5Y-51.0	High risk (>3)	4Y-38.4	4Y-14.3
	Stage III/IV	High risk (≥6)	5Y-33.0	5Y-50.0			
	EN sites≥1						
PIT N=385	Age>60 years	Low risk (0)	5Y-58.9	NA			
	ECOG PS ≥2	Low-intermediate (1)	5Y-45.6	NA	Low risk (≤2)	4Y-48.3	4Y-19.4
	LDH>ULN	High-intermediate (2)	5Y-39.7	NA			
	Bone marrow invasion	High risk (3-4)	5Y-18.3	NA	High risk (>2)	4Y-40.8	4Y-24.0

OS, Overall survival; PFS, progression-free survival; AITL, angioimmunoblastic T-cell lymphoma; IPI, International prognostic index; NCCN-IPI, National Comprehensive Cancer Network; PIAI, the alternative Prognostic Index for AITL; PIT, Prognostic Index for T-cell lymphoma Prognostic index for AITL; ATPI, AITL Prognostic Index; AITL score, angioimmunoblastic T-cell lymphoma prognostic index; Chinese AITL score, prognostic. index of angioimmunoblastic T-cell lymphoma in China; ECOG PS, Eastern Cooperative Oncology Group performance score; LDH, lactic dehydrogenase; WBC, white blood cell; Hb, hemoglobin; PLT, platelet; Ig, immunoglobulin; ULN, upper limit of normal; EN, extranodal site involvement; extranodal site involvement of bone marrow, central nervous system, lung, liver or gastrointestinal tract.; NA, not assessed.

## 4 Discussion

AITL originates from TFH cells and is characterized by distinct clinical, morphological, immunophenotypic, and genomic features. AITL cells not only express common antigenic markers associated with T cells, such as CD3,CD7 and CD4 but also exhibit specific markers indicative of TFH cells, including CD10, CXCL13, ICOS, and PD-1 (29). Nonetheless, these immunological markers are mostly nonspecific (30). BCL6, CD10, and CXCL13 exhibit good specificity but relatively low sensitivity in the diagnosis of AITL (31). It is generally held that the pathological diagnosis of AITL requires the positivity of at least two or three TFH cell markers, in conjunction with a thorough comprehensive judgment of the tissue morphology. The high percentages of PD-1-, PD-L1-, BCL6-, CXCL13-, CD30-, and CD7-positive cells observed in our cohort are consistent with previous literature (32, 33). Moreover, multivariate analysis in our study indicated that age over 70 years, bone marrow involvement, and CD7 negativity are independent risk factors negatively affecting PFS. This suggests that CD7 may serve as one of the predictive indicators for long-term prognosis of AITL.

There is no established standard of care for AITL. The therapeutic options of AITL are similar to those for other PTCL subtypes. First-line treatments frequently include CHOP-like regimens, but the prognosis is generally poor (34). Therefore, researchers are actively testing methods to improve the efficacy of first-line treatment for AITL. The outcomes of these efforts have been varied. Schmitz et al. (35) found that the addition of etoposide to the CHOP regimen did not improve PFS in patients with PTCL compared to CHOP alone. Subgroup analysis showed that only anaplastic lymphoma kinase (ALK)-positive anaplastic large-cell lymphoma (ALCL) patients benefited from the CHOPE regimen. Sun et al. (36) conducted a trial in newly diagnosed PTCL patients comparing the GDPT regimen with the standard CHOP regimen, which showed that the GDPT regimen demonstrated superior ORR and CR rates, as well as PFS and OS. In our study, the CHOP-like regimen led to marginally better CR and ORR rates, along with a longer DOR, than the non-CHOP regimen. We believe that the CHOP-like regimens remain the preferred first-line treatment option for AITL.

Furthermore, advancements in understanding the tumor microenvironment have revealed that histone deacetylase (HDAC) inhibitor chidamide may emerge as a potentially beneficial agent for AITL patients. A multicenter, real-world study indicated that chidamide, when combined with chemotherapy, improved treatment efficacy in patients with relapsed or refractory AITL (28). However, our study’s findings indicate that the incorporation of chidamide in first-line induction and maintenance treatment did not lead to higher remission rates or longer DOR. A separate phase III study also reported that incorporation of CHOP with romidepsin, another HDAC inhibitor, did not improve PFS, response rates, or OS compared with standard CHOP and was associated with higher risk of grade ≥3 adverse events (37). Prospective, randomized, controlled clinical trials will be needed to evaluate the efficacy and safety of HDAC inhibitors in combination with chemotherapy as first-line treatment for AITL. This would help to establish the role of HDAC inhibitors in this field.

ASCT has traditionally been considered as the standard consolidation therapy for patients with PTCL who achieve CR1 or PR1. However, A study of 160 newly diagnosed PTCL patients revealed that after responding to induction chemotherapy with the CHOP regimen and undergoing ASCT consolidation, there was no significant long-term survival benefit (38). In our research, we observed no extension in DOR for chemo-sensitive patients who received first-line ASCT consolidation. The absence of a significant benefit could be attributed to the small sample size of patients undergoing ASCT. Factors contributing to this low number include the advanced age of our cohort, with a median of 62 years and over 50% being above 60, and the limited prevalence and acceptance of ASCT in China. Many institutions lack the capacity to perform ASCT, and a significant number of patients are hesitant to opt for it. Despite these challenges, we believe ASCT has the potential to enhance patient outcomes and encourage its consideration for eligible patients in CR1.

Finally, in this study, we conducted a comprehensive evaluation and comparison of seven prognostic models. Our findings indicate that while all the models can accurately predict the prognosis of high- and low-risk AITL patients, their performance varies significantly. Comparing the discriminatory power of these seven models, it became evident that the AITL score, PIAI score, and Chinese AITL score were superior, showing higher C-index values. When considering discriminability, feasibility, and repeatability, the Chinese AITL score outperformed the other scores. Therefore, we recommend Chinese AITL score as the basis for AITL risk stratification in future research.

CD7, a co-stimulatory receptor protein that aids in T cell activation, is commonly expressed on approximately 85% of peripheral T cells. This molecule has the characteristic of undergoing endocytosis upon binding with antibodies and their derivatives, making it an ideal target for immunotoxin therapy. Immunotoxins can bind to CD7, be internalized by cells, and exert their cytotoxic effects intracellularly (39, 40). Studies have shown that in acute myeloid leukemia (AML), the positive expression of CD7 is an independent unfavorable prognostic factor for PFS (41). However, our study revealed that the loss of CD7 expression is associated with worse prognosis of AITL patients, which is interesting and deserves further studies.

The scoring model presented in this study integrates common and clinically relevant factors such as age and bone marrow involvement, and for the first time, includes the pathological marker CD7. Our model has demonstrated robust discriminative power and has been successfully validated in an independent cohort. However, this study has several key methodological limitations. First, the absence of a standardized central pathology review mechanism has led to inter-center heterogeneity in critical technical parameters, including antibody selection (particularly for CD7 detection), immunohistochemical staining protocols, and interpretation criteria. Such technical variability, compounded by batch-to-batch antibody differences and subjective staining intensity assessments, may systematically bias biomarker reliability. Second, the validation cohort’s limited sample size, especially in subgroup analyses, reduces statistical power and risks obscuring clinically relevant differences, necessitating validation through expanded cohorts and multi-source data integration. Third, inter-observer variability in quantitative staining evaluation introduces additional interpretive uncertainty. Fourth, retrospective inconsistencies in documenting CHOP-like regimen dosing across institutions, particularly unrecorded dose adjustments, introduce potential confounding. While all cases adhered to the core drug combination (cyclophosphamide, doxorubicin, vincristine, prednisone) and 21-day cycle framework, dose variations within guideline-permitted thresholds may still contribute to outcome heterogeneity.

Notably, the confluence of technical inconsistencies, sample size constraints, and undocumented therapeutic variability may collectively compromise the accuracy of CD7’s predictive biomarker evaluation. Given these compounded limitations, current conclusions regarding CD7’s predictive utility require guarded interpretation. Future multicenter investigations should implement four critical improvements: 1) protocol-driven central pathology review systems with quality-controlled antibody panels; 2) prospective large-scale cohorts with pre-specified subgroup power calculations; 3) standardized digital pathology platforms for quantitative staining analysis; and 4) rigorous documentation of therapeutic parameters, including drug dose adjustments, to minimize confounding. These refinements will enhance both reproducibility and clinical translatability. Finally, given that the Chinese AITL scoring system has demonstrated excellent performance in predicting both OS and PFS, we recommend its adoption as the preferred prognostic assessment tool for Chinese patients with AITL.

## 5 Conclusion

This retrospective analysis of 140 AITL patients’ real-world data from the South China T-cell Lymphoma Collaborative Group indicates that the prognosis for AITL patients under first-line CHOP and ASCT treatment remains unfavorable. The integration of novel agents, such as chidamide, with first-line therapy was found to be less than optimal. The Chinese AITL score demonstrated robust predictive power for long-term patient outcomes. Additionally, pathologic biomarkers, like loss of CD7 expression, are gaining traction as new prognostic indicators. Meanwhile, our novel prognostic model combining the abovementioned three factors may improve the risk stratification for AITL. Given the study’s limited sample size and retrospective design, there is a clear need for larger, multicenter, and prospective clinical trials to advance the understanding and treatment of AITL.

## Data Availability

The original contributions presented in the study are included in the article/**Supplementary Material**. Further inquiries can be directed to the corresponding authors.

## References

[1] Vose J Armitage J and DW. International peripheral T-cell and natural killer/T-cell lymphoma study: pathology findings and clinical outcomes. J Clin Oncol. (2008) 26:4124–30. doi: 10.1200/jco.2008.16.4558 18626005

[2] Ferry JA. Angioimmunoblastic T-cell lymphoma. Adv Anat Pathol. (2002) 9:273–9. doi: 10.1097/01.PAP.0000026242.53167.A5 12195216

[3] Quivoron C Couronné L Della Valle V Lopez CK Plo I Wagner-Ballon O. TET2 inactivation results in pleiotropic hematopoietic abnormalities in mouse and is a recurrent event during human lymphomagenesis. Cancer Cell. (2011) 20:25–38. doi: 10.1016/j.ccr.2011.06.003 21723201

[4] Couronné L Bastard C and OAB. TET2 and DNMT3A mutations in human T-cell lymphoma. N Engl J Med. (2012) 366:95–6. doi: 10.1056/NEJMc1111708 22216861

[5] Sakata-Yanagimoto M Enami T Yoshida K Shiraishi Y Ishii R Miyake Y. Somatic RHOA mutation in angioimmunoblastic T cell lymphoma. Nat Genet. (2014) 46:171–5. doi: 10.1038/ng.2872 24413737

[6] Cairns RA Iqbal J Lemonnier F Kucuk C de Leval L Jais JP. IDH2 mutations are frequent in angioimmunoblastic T-cell lymphoma. Blood. (2012) 119:1901–3. doi: 10.1182/blood-2011-11-391748 PMC3293643 22215888

[7] Wang C McKeithan TW Gong Q Zhang W Bouska A Rosenwald A. IDH2R172 mutations define a unique subgroup of patients with angioimmunoblastic T-cell lymphoma R172 mutations define a unique subgroup of patients with angioimmunoblastic T-cell lymphoma. Blood. (2015) 126:1741–52. doi: 10.1182/blood-2015-05-644591 PMC4600014 26268241

[8] Figueroa ME Abdel-Wahab O Lu C Ward PS Patel J Shih A. Leukemic IDH1 and IDH2 mutations result in a hypermethylation phenotype, disrupt TET2 function, and impair hematopoietic differentiation. Cancer Cell. (2010) 18:553–67. doi: 10.1016/j.ccr.2010.11.015 PMC4105845 21130701

[9] Lemonnier F Cairns RA Inoue S Li WY Dupuy A Broutin S. The IDH2 R172K mutation associated with angioimmunoblastic T-cell lymphoma produces 2HG in T cells and impacts lymphoid development. Proc Natl Acad Sci U S A. (2016) 113:15084–9. doi: 10.1073/pnas.1617929114 PMC5206549 27956631

[10] Wei C Li W Qin L Liu S Xue C Ren K. Clinicopathologic characteristics, outcomes, and prognostic factors of angioimmunoblastic T-cell lymphoma in China. Cancer Med. (2023) 12:3987–98. doi: 10.1002/cam4.5248 PMC9972121 36106610

[11] Advani RH Skrypets T Civallero M Spinner MA Manni M Kim WS. Outcomes and prognostic factors in angioimmunoblastic T-cell lymphoma: final report from the international T-cell Project. Blood. (2021) 138:213–20. doi: 10.1182/blood.2020010387 PMC8493974 34292324

[12] Horwitz SM Ansell S Ai WZ Barnes J Barta SK Brammer J. T-cell lymphomas, version 2. 2022 NCCN Clin Pract Guidelines Oncol J Natl Compr Canc Netw. (2022) 20:285–308. doi: 10.6004/jnccn.2022.0015 35276674

[13] Horwitz S O’Connor OA Pro B Trümper L Iyer S Advani R. The ECHELON-2 Trial: 5-year results of a randomized, phase III study of brentuximab vedotin with chemotherapy for CD30-positive peripheral T-cell lymphoma. Ann Oncol. (2022) 33:288–98. doi: 10.1016/j.annonc.2021.12.002 PMC9447792 34921960

[14] Camus V Thieblemont C Gaulard P Trümper L Iyer S Advani R. Romidepsin plus cyclophosphamide, doxorubicin, vincristine, and prednisone versus cyclophosphamide, doxorubicin, vincristine, and prednisone in patients with previously untreated peripheral T-cell lymphoma: final analysis of the ro-CHOP trial. J Clin Oncol. (2024) 42:1612–8. doi: 10.1200/JCO.23.01687 38364196

[15] Shi Y Dong M Hong X Zhang W Feng J Zhu J. Results from a multicenter, open-label, pivotal phase II study of chidamide in relapsed or refractory peripheral T-cell lymphoma. Ann Oncol. (2015) 26:1766–71. doi: 10.1093/annonc/mdv237 26105599

[16] Wang J Su N Fang Y Ma S Zhang Y Cai J. Comparison of chemotherapy combined with chidamide versus chemotherapy in the frontline treatment for peripheral T-cell lymphoma. Front Immunol. (2022) 13:835103. doi: 10.3389/fimmu.2022.835103 35185926 PMC8847145

[17] Mehta-Shah N Kommalapati A Teja S Cashen AF Dahi PB Sauter CS. Successful treatment of mature T-cell lymphoma with allogeneic stem cell transplantation: the largest multicenter retrospective analysis. Blood. (2020) 136:5. doi: 10.1182/blood-2020-138542

[18] Swerdlow SH Campo E Pileri SA Harris NL Stein H Siebert R. The 2016 revision of the World Health Organization classification of lymphoid neoplasms. Blood. (2016) 127:2375–90. doi: 10.1182/blood-2016-01-643569 PMC4874220 26980727

[19] Cheson BD Fisher RI Barrington SF Cavalli F Schwartz LH Zucca E. Recommendations for initial evaluation, staging, and response assessment of hodgkin and non-hodgkin lymphoma: the lugano classification. J Clin Oncol. (2014) 32:3059–67. doi: 10.1200/jco.2013.54.8800 PMC4979083 25113753

[20] National Comprehensive Cancer Network NCCN clinical practice guidelines in oncology (NCCN guidelines^®^) T-cell lymphomas version 2.2024. [EB/OL] (2024). Available online at: https://www.nccn.org/guidelines/ (Accessed June 5, 2024).

[21] Project I N-H s L P F. A predictive model for aggressive non-Hodgkin’s lymphoma. N Engl J Med. (1993) 329:987–94. doi: 10.1056/NEJM199309303291402 8141877

[22] Gallamini A Stelitano C Calvi R Bellei M Mattei D Vitolo U. Peripheral T-cell lymphoma unspecified (PTCL-U): a new prognostic model from a retrospective multicentric clinical study. Blood. (2004) 103:2474–9. doi: 10.1182/blood-2003-09-3080 14645001

[23] Zhou Z Sehn LH Rademaker AW Gordon LI Lacasce AS Crosby-Thompson A. An enhanced International Prognostic Index (NCCN-IPI) for patients with diffuse large B-cell lymphoma treated in the rituximab era. Blood. (2014) 123:837–42. doi: 10.1182/blood-2013-09-524108 PMC5527396 24264230

[24] Tokunaga T Shimada K Yamamoto K Chihara D Ichihashi T Oshima R. Retrospective analysis of prognostic factors for angioimmunoblastic T-cell lymphoma: a multicenter cooperative study in Japan. Blood. (2012) 119:2837–43. doi: 10.1182/blood-2011-08-374371 22308294

[25] Federico M Rudiger T Bellei M Nathwani BN Luminari S Coiffier B. Clinicopathologic characteristics of angioimmunoblastic T-cell lymphoma: analysis of the international peripheral T-cell lymphoma project. J Clin Oncol. (2013) 31:240–6. doi: 10.1200/jco.2011.37.3647 PMC3532394 22869878

[26] Tripepi G Jager KJ Dekker FW Zoccali C. Statistical methods for the assessment of prognostic biomarkers (Part I): Discrimination. Nephrol Dial Transplant. (2010) 25:1399–401. doi: 10.1093/ndt/gfq018 20139066

[27] Uno H Cai T Pencina MJ D'Agostino RB Wei LJ. On the C-statistics for evaluating overall adequacy of risk prediction procedures with censored survival data. Stat Med. (2011) 30:1105–17. doi: 10.1002/sim.4154 PMC3079915 21484848

[28] Ahearne MJ Allchin RL Fox CP Wagner SD. Follicular helper T-cells: expanding roles in T-cell lymphoma and targets for treatment. Br J Haematol. (2014) 166:326–35. doi: 10.1111/bjh.12941 24815671

[29] Yoon SE Cho J Kim YJ Ko YH Park WY Kim SJ. Comprehensive analysis of clinical, pathological, and genomic characteristics of follicular helper T-cell derived lymphomas. Exp Hematol Oncol. (2021) 10:33. doi: 10.1186/s40164-021-00224-3 33990228 PMC8120779

[30] Yi X Jaffe ES. How I diagnose angioimmunoblastic T-cell lymphoma. Am J Clin Pathol. (2021) 156:1–14. doi: 10.1093/ajcp/aqab090 34117736 PMC8209595

[31] Chiba S Sakata-Yanagimoto M. Advances in understanding of angioimmunoblastic T-cell lymphoma. Leukemia. (2020) 34:2592–606. doi: 10.1038/s41375-020-0990-y PMC7376827 32704161

[32] Bossard C Dobay MP Parrens M Lamant L Missiaglia E Haioun C. Immunohistochemistry as a valuable tool to assess CD30 expression in peripheral T-cell lymphomas: high correlation with mRNA levels. Blood. (2014) 124:2983–6. doi: 10.1182/blood-2014-07-584953 25224410

[33] Watanabe N Mo F Zheng R Ma R Bray VC van Leeuwen DG. Feasibility and preclinical efficacy of CD7-unedited CD7 CAR T cells for T cell Malignancies. Mol Ther. (2023) 31:24–34. doi: 10.1016/j.ymthe.2022.09.003 36086817 PMC9840107

[34] Mohammed Saleh MF Kotb A Abdallah GEM Muhsen IN El Fakih R Aljurf M. Recent advances in diagnosis and therapy of angioimmunoblastic T cell lymphoma. Curr Oncol. (2021) 28:5480–98. doi: 10.3390/curroncol28060456 PMC8699908 34940095

[35] Schmitz N Trümper L Ziepert M Nickelsen M Ho AD Metzner B. Treatment and prognosis of mature T-cell and NK-cell lymphoma: an analysis of patients with T-cell lymphoma treated in studies of the German High-Grade Non-Hodgkin Lymphoma Study Group. Blood. (2010) 116:3418–25. doi: 10.1182/blood-2010-02-270785 20660290

[36] Sun Y Li L Li X Zhang L Wang X Fu X. Outcomes of GDPT (gemcitabine, cisplatin, prednisone, thalidomide) versus CHOP in newly diagnosed peripheral T-cell lymphoma patients. Ther Adv Med Oncol. (2020) 12:1758835920923829. doi: 10.1177/1758835920923829 32550864 PMC7278096

[37] Bachy E Camus V Thieblemont C Sibon D Casasnovas RO Ysebaert L. Romidepsin plus CHOP versus CHOP in patients with previously untreated peripheral T-cell lymphoma: results of the ro-CHOP phase III study (Conducted by LYSA). J Clin Oncol. (2022) 40:242–51. doi: 10.1200/JCO.21 34843406

[38] d’Amore F Relander T Lauritzsen GF Jantunen E Hagberg H Anderson H. Up-front autologous stem-cell transplantation in peripheral T-cell lymphoma: NLG-T-01. J Clin Oncol. (2012) 30:3093–9. doi: 10.1038/s41375-022-01545-w 22851556

[39] Tang J Li J Zhu X Yu Y Chen D Yuan L. Novel CD7-specific nanobody-based immunotoxins potently enhanced apoptosis of CD7-positive Malignant cells. Oncotarget. (2016) 7:34070–83. 10.18632/oncotarget.8710 PMC5085138 27083001

[40] Pauza ME Doumbia SO and CAP. Construction and characterization of human CD7-specific single-chain Fv immunotoxins. J Immunol. (1997) 158:3259–69. doi: 10.4049/jimmunol.158.7.3259 9120282

[41] Liu J Zhang Y Guo R Zhao Y Sun R Guo S. Targeted CD7 CAR T-cells for treatment of T-Lymphocyte leukemia and lymphoma and acute myeloid leukemia: recent advances. Front Immunol. (2023) 14:1170968. doi: 10.3389/fimmu.2023.1170968 37215124 PMC10196106

