# Supplementary material for: Real-world data on clinical outcomes and validation of prognostic models for angioimmunoblastic T-cell lymphoma: a multicentric retrospective study in Southern China
Source: Front Oncol. 2025 Jun 10;15:1580370. doi: 10.3389/fonc.2025.1580370 (PMC12185395; doi:10.3389/fonc.2025.1580370)
Supplement: Supplementary file 1 [file Table1.docx]

| **Supplementary Table1**  **Pathological characteristic** | |
| --- | --- |
| Pathological | No. (%) |
| CD3 positive | 130/131(99.24) |
| CD4 positive | 102/103(99.03) |
| CD5 positive | 98/102(96.08) |
| CD7 positive | 57/71(80.28) |
| CD8 positive | 63/91(69.23) |
| CD10 positive | 74/117(63.25) |
| CD23 positive | 26/36(72.22) |
| CD30 positive | 79/101(78.22) |
| CD56 positive | 11/52(21.15) |
| TdT positive | 3/14(21.43) |
| CXCL-13 positive | 49/57(85.96) |
| BCL-6 positive | 74/95(77.89) |
| PD-1 positive | 81/91(89.01) |
| PDL-1 positive | 10/14(71.43) |
| TIA-1 positive | 31/49(63.27) |
| GrB positive | 17/39(43.59) |
| ALK positive | 3/45(6.67) |
| EBER positive | 64/101(63.37) |
| TP63 positive | 0/2(0.00) |
| DUSPP22-IRF4 positive | 0/5(0.00) |
| TP63 rearrangement positive | 0/5(0.00) |
| NPM-ALK positive | 0/5(0.00) |
