# Supplementary material for: Real-world data on clinical outcomes and validation of prognostic models for angioimmunoblastic T-cell lymphoma: a multicentric retrospective study in Southern China
Source: Front Oncol. 2025 Jun 10;15:1580370. doi: 10.3389/fonc.2025.1580370 (PMC12185395; doi:10.3389/fonc.2025.1580370)
Supplement: Supplementary file 2 [file Table2.docx]

| **Supplementary Table2 Univariate comparison between fist-line chemotherapy with CHOP-like regimens or non-chop-like regimens** | | | | | |
| --- | --- | --- | --- | --- | --- |
| **Variable** | **CHOP-like (n=116, %)** | | **Non-CHOP-like (n=24, %)** | **P value** | |
| **Gender** | | | | 0.498 | |
| Man | 72(62.1) | | 13(54.2) |  | |
| Woman | 44(37.9) | | 11(45.8) |  | |
| **Age1** | | | | 0.502 | |
| ≤60 years | 54(46.6) | | 9(37.5) |  | |
| >60 years | 62(53.4) | | 15(62.5) |  | |
| **Age2** | | | | 0.002 | |
| ≤70 years | 104(89.7) | | 15(62.5) |  | |
| >70 years | 12(10.3) | | 9(37.5) |  | |
| **Ann Arbor** | | | | 0.360 | |
| <III stage | 9(8.0) | | 0(0.0) |  | |
| ≥III stage | 104(92.0) | | 24(100.0) |  |  |
| **Extranodal involvement1** | | | | 1.000 | |
| ≤1 | 92(79.3) | | 19(79.2) |  | |
| >1 | 24(20.7) | | 5(20.8) |  | |
| **Extranodal involvement2** | | | | 1.000 | |
| <2 | 8(88.9) | | 1(100.0) |  | |
| ≥2 | 1(11.1) | | 0(0.0) |  | |
| Unkown |  | |  |  | |
| **WBC** | | | | 0.314 | |
| ≤10×10*9 | 83(72.2) | | 20(83.3) |  | |
| >10×10*9 | 32(27.8) | | 4(16.7) |  | |
| **HB** | | | | 0.810 | |
| <100g/L | 83(72.2) | | 18(75.0) |  | |
| ≥100g/L | 32(27.8) | | 6(25.0) |  | |
| **PLT** | | | | 0.468 | |
| <150×10*9/L | 82(71.3) | | 19(79.2) |  | |
| ≥150×10*9/L | 33(28.7) | | 5(20.8) |  | |
| **LDH** | | | | 0.490 | |
| ≤240U/L | 42(36.8) | | 11(45.8) |  | |
| >240U/L | 72(63.2) | | 13(54.2) |  | |
| **CRP** | | | | 0.776 | |
| <10mg/L | 24(37.5) | | 5(31.3) |  | |
| ≥0mg/L | 40(62.5) | | 11(68.8) |  | |
| Unkown |  | |  |  | |
| **Bone marrow involvement** | | | | 0.409 | |
| Yes | 21(18.1) | | 6(25.0) |  | |
| No | 95(81.9) | | 18(75.0) |  | |
| **Albumin** | | | | 0.822 | |
| <35g/L | 65(57.5) | | 13(54.2) |  | |
| ≥35g/L | 48(42.5) | | 11(45.8) |  | |
| **IgA** | | | | 1.000 | |
| ≤400 mg/dl | 30(76.9) | | 5(83.3) |  | |
| >400mg/dl | 9(23.1) | | 1(16.7) |  | |
| Unkown |  | |  |  | |
| **ECOG PS1** | | | | 0.509 | |
| ≤1 | 70(74.5) | | 13(65.0) |  | |
| >1 | 23(24.5) | | 7(35.0) |  | |
| **ECOG PS2** | | | | 0.287 | |
| ≤2 | 89(95.7) | | 18(90.0) |  | |
| >2 | 4(4.3) |  | 2(10.0) |  | |
| **Ki-67** | | | | 0.626 | |
| ≤60% | 68(65.4) | | 15(71.4) |  | |
| >60% | 36(34.6) | | 6(28.6) |  | |
| **β2-microglobulin** | | | | 0.067 | |
| ≤2.4mg/l | 18(23.1) | | 1(19.2) |  | |
| >2.4mg/l | 60(76.9) | | 20(95.2) |  | |
|  |  | |  |  | |

Note: WBC, white blood cell; HB, hemoglobin; PLT, platelet; Ig, immunoglobulin; LDH, lactate dehydrogenase; CRP, Hypersensitivity proteins; ECOG, Eastern Cooperative Oncology Group; WBC, white blood cell.
