# Supplementary material for: Real-world data on clinical outcomes and validation of prognostic models for angioimmunoblastic T-cell lymphoma: a multicentric retrospective study in Southern China
Source: Front Oncol. 2025 Jun 10;15:1580370. doi: 10.3389/fonc.2025.1580370 (PMC12185395; doi:10.3389/fonc.2025.1580370)
Supplement: Supplementary file 3 [file Table3.docx]

| **Supplementary Table3 Univariate comparison between fist-line chemotherapy in combination with and without chidamide** | | | |  |
| --- | --- | --- | --- | --- |
| **Variable** | **With chidamide (n=89, %)** | **Without chidamide (n=51, %)** | **P value** |  |
| **Gender** | | | **0.479** |  |
| Man | 52 (58.4) | 33(64.7) |  |  |
| Woman | 37(41.6) | 18(35.3) |  |  |
| **Age1** | | | **0.486** |  |
| ≤60 years | 38(46.6) | 25(37.5) |  |  |
| >60 years | 51(53.4) | 26(62.5) |  |  |
| **Age2** | | | **0.325** |  |
| <70 years | 78(87.6) | 41(80.4) |  |  |
| >70 years | 11(12.4) | 10(19.6) |  |  |
| **Ann Arbor** | | | **0.486** |  |
| <III stage | 7(8.0) | 2(4.0) |  |  |
| >III stage | 80(92.0) | 48(96.0) |  |  |
| **Extranodal involvement1** | | | **0.082** |  |
| ≤1 | 75(84.3) | 36(70.6) |  |  |
| >1 | 14（15.7) | 15(29.4) |  |  |
| **Extranodal involvement2** | | | **1.000** |  |
| <2 | 7(87.5) | 2(100.0) |  |  |
| ≥2  Unknow | 1(12.5) | 0(0.0) |  |  |
| **WBC** | | | **1.000** |  |
| <10×10*9 | 66(74.2) | 37(74.0) |  |  |
| >10×10*9 | 23(25.8) | 13(26.0) |  |  |
| **HB** | | | **0.845** |  |
| <100g/L | 64(71.9) | 37(74.0) |  |  |
| >100g/L | 25(28.1) | 13(26.0) |  |  |
| **PLT** | | | **0.557** |  |
| <150×10*9/L | 63(70.8) | 38(76.0) |  |  |
| >150×10*9/L | 26(29.2) | 12(24.0) |  |  |
| **LDH** | | | **0.069** |  |
| ≤240U/L | 29(32.6) | 24(49.0) |  |  |
| >240U/L | 60(67.4) | 25(51.0) |  |  |
| **CRP** | | | **0.240** |  |
| <10mg/L | 21(41.2) | 8(27.6) |  |  |
| ≥10mg/L | 30(58.8) | 21(72.4) |  |  |
| **Bone marrow involvement** | | | **0.377** |  |
| No | 74(83.1) | 39(76.5) |  |  |
| Yes | 15(16.9) | 12(23.5) |  |  |
| **Albumin** | | | **0.154** |  |
| <35g/L | 46(52.3) | 32(65.3) |  |  |
| >35g/L | 42(47.7) | 17(34.7) |  |  |
| **IgA** | | | **0.292** |  |
| ≤400 mg/dl | 21(72.4) | 14(87.5) |  |  |
| >400mg/dl  Unknow | 8(27.6) | 2(12.5) |  |  |
| **ECOG PS1** | | | **0.604** |  |
| ≤1 | 55(75.3) | 28(68.3) |  |  |
| >1 | 17(23.3) | 13(31.7) |  |  |
| **ECOG PS2** | | | **0.666** |  |
| ≤2分 | 69(95.8) | 38(92.7) |  |  |
| >2分 | 3(4.2) | 3(7.3) |  |  |
| **Ki-67** | | | **0.560** |  |
| <60% | 50(64.1) | 33(70.2) |  |  |
| >60% | 28(35.9) | 14(29.8) |  |  |
| **β2-microglobulin** | | | **0.200** |  |
| <2.4mg/l | 14(23.7) | 5(12.5) |  |  |
| >2.4mg/l | 45(76.3) | 35(87.5) |  |  |

Note: WBC, white blood cell; HB, haemoglobin; PLT, platelet; LDH, lactate dehydrogenase; CRP, C-reactive protein.
